# The influence of socio-economic and surveillance characteristics on breast cancer survival: a French population-based study

**DOI:** 10.1038/sj.bjc.6604163

**Published:** 2008-01-08

**Authors:** J Gentil-Brevet, M Colonna, A Danzon, P Grosclaude, G Chaplain, M Velten, F Bonnetain, P Arveux

**Affiliations:** 1 Registre des Cancers du Sein et autres Cancers Gynécologiques de Côte d'Or, Centre Georges-François Leclerc, 1 rue du Professeur Marion—BP 77980, Dijon Cedex 21079, France; 2 EA 4184, Université de Bourgogne, Dijon 21000, France; 3 Registre des Cancers de l'Isère, 23 Chemin des Sources, Meylan 38240, France; 4 Registre des Cancers du Doubs, CHU Saint Jacques, Besançon Cedex 25030, France; 5 Registre des Cancers du Tarn, Albi 81000, France; 6 Registre des Cancers du Bas-Rhin, Centre de Lutte Contre le Cancer Paul Strauss, Strasbourg 67000, France

**Keywords:** breast neoplasm, mammography, mass screening, survival analysis, socio-economic factors

## Abstract

Survival data on female invasive breast cancer with 9-year follow-up from five French cancer registries were analysed by logistic regression for prognostic factors of cancer stage. The Kaplan–Meier method and log-rank test were used to estimate and compare the overall survival probability at 5 and 7 years, and at the endpoint. The Cox regression model was used for multivariate analysis. County of residence, age group, occupational status, mammographic surveillance, gynaecological prevention consultations and the diagnosis mammography, whether within a screening framework or not, were independent prognostic factors of survival. Moreover, for the same age group, and only for cancers T2 and/or N+ (whether 1, 2 or 3) and M0, the prognosis was significantly better when the diagnosis mammography was done within the framework of screening. Socio-economic and surveillance characteristics are independent prognostic factors of both breast cancer stage at diagnosis and of survival. Screening mammography is an independent prognostic factor of survival.

Breast cancer is the commonest type of cancer among women worldwide, accounting for approximately 20% of all malignancy and higher in developed western countries (Boyle and Ferlay, 2004; Parkin *et al*, 2005); its incidence has been rising in the United States, Canada, Europe, Singapore and Japan. Many prognostic factors are well described, but socio-economic factors and screening mammography have produced contradictory results (Klemi *et al*, 2003; Gill *et al*, 2004; Joensuu *et al*, 2004). In France, population-based cancer survival data collected by cancer registries are useful in assessing the effectiveness of strategies to control cancer incidence (Chia *et al*, 2001), but only the Côte d'Or breast and gynaecologic cancer registry focuses on breast and gynaecologic cancer. It has been collecting comprehensive population-based data since 1982.

In this study, we have investigated socio-economic and surveillance characteristics as prognostic factors for breast cancer stage at diagnosis and overall survival. We also examined screening mammography as a prognostic factor for overall survival of patients with the same stage of breast cancer at diagnosis, and of patients in the same age group.

## MATERIALS AND METHODS

### Patients

A sample of 1150 women diagnosed with invasive breast cancer in 1995–1997 was followed up in 2006 to examine the relation of socio-economic and surveillance characteristics to survival. This sample was built from five population-based cancer registries in France and included the following: cases of all breast cancer diagnosed in incidents in the counties of Doubs (*n*=248) and Tarn (*n*=212) in 1997, Côte d'Or (*n*=228) over 10 months of 1997 and as was a random sample of cases in the counties of Bas-Rhin (*n*=193) in 1995 and Isère (*n*=269) in 1997; *in situ* breast cancer and cases in men were not included.

For all patients, we gathered follow-up data such as life status, last news or date of death, the date of any relapse or metastasis of breast cancer and type and place of treatment. Follow-up questionnaires were completed by the registries and centralized in the Côte d'Or registry, where the data were entered into a database. The registries ascertained the life status of patients from various sources (e.g., RNIPP (Répertoire National d'Identification des Personnes Physiques), town hall of place of birth, death certificates, medical records, general practitioner or CNAM (Caisse Nationale d'Assurance Maladie)). Clinical details and treatments were obtained from clinical records, but when these were incomplete, the missing data were obtained from the general practitioner or the specialist using a questionnaire.

The variable ‘socio-economic status’ was determined from occupational status and, when data were missing, from the level of education. The occupational status was split into two classes: low or middle (farmers, artisans, manual workers, unemployed) and high (executives, middle professional group, clerical employees). The education level was also split into two classes: low (no diploma, certificate of primary education, low-level vocational certificate, low-level professional diploma) and high (high-school diploma, higher education). The socio-economic status was low or high when the occupational status was low or high, respectively, and if the latter was unknown, the socio-economic status was low or high when the education level was low or high, respectively.

The stage of the primary breast cancer was determined using the TNM staging system for breast cancer and classified into one of four categories as follows: stage 1, T1a and T1b N0 M0; stage 2, T1c N0 M0; stage 3, T2 and/or N+ (whether 1, 2 or 3) M0 and stage 4, T3 and 4 and/or M1, whatever the N (0–3).

The five circumstances of diagnostic mammography reduced to two categories: screening or no screening. The first includes mammography screening programmes and symptom-free screening mammography, and the second includes mammographies performed on clinical symptoms observed by the doctor or the woman herself, or for women with a high-risk for breast cancer.

### Statistical analysis

The statistical analysis was carried out at the Côte d'Or breast cancer registry. Continuous and qualitative variables were respectively described by mean, standard deviation, median and percentage. The percentage of missing values was also provided. Univariate and multivariate analyses of prognostic factors for stage 1 (T1a or T1b N0 M0) cancer *vs* all other stages together were carried out using logistic regression, and the significance level *α*=0.05 was used to select variables for the multivariate model. The following variables were included: place of residence (Bas-Rhin, Côte d'Or, Doubs, Isère, Tarn), age at diagnosis (<50 years old, 50–70 years old, >70 years old), way of life according to marital status (alone or not), number of children (0 or 1, 2 or more); occupational status was divided into two categories, as described above, socio-economic status (low or high, according to occupational status and education), number of mammographies during the 6 years before diagnosis (none or at least one), gynaecological prevention consultations during the 3 years before diagnosis (none or at least one). All variables were included in the multivariate model to calculate the odds ratio (OR) and its 95% confidence interval (CI). The correlation between these variables was tested beforehand to explore associations and multi-collinearity.

The follow-up period continued from the date of diagnosis until death or until June 30, 2006 (data cut-off) if patients were still living. Overall survival, measured from diagnosis until death (all causes) or last follow-up, was estimated using the Kaplan–Meier method, and survival curves were compared using log-rank tests. For multivariate analysis, the Cox regression model was applied and the variables analysed were the same as those in the study of prognostic factors by stage at diagnosis, cancer detected by screening mammography or not. Hazard ratios (HRs) and their 95% CIs were calculated. Multivariate survival analysis was first based on significant factors for stage at diagnosis, and then using stage in the same model. Univariate analyses of survival according to the circumstances of the diagnosis mammography, including age and clinical stage subgroups (*post hoc* analysis), were performed. For clinical stage subgroups, our original intention was to analyse the impact of screening on mortality for each stage separately, but women with stage 1 (T1a or T1b N0 M0) at diagnosis had much higher survival: among 158 patients, only three died within 5 years of diagnosis, and so this was combined with stage 2 (T1c N0 M0) subgroup in the analysis. The same analysis was performed after separating stage 3 (T2 N0/+ M0) into three classes: T1 N+ M0, T2 N0 M0 and T2 N+ M0. All calculations were carried out using SAS v 9.1 software and the significance level *α*=0.05 was used.

## RESULTS

The patients' socio-economic and surveillance characteristics are shown in Table 1. The mean age of patients at diagnosis was 59.9 years (95% CI=[33.2–86.7]), and was not significantly different according to the county of residence; 280 (24.3%) were under 50 years old, 569 (49.5%) were 50–70 years old and 292 (25.4%) were over 70 years old. Three hundred and fifty-four women were living alone (30.8%), while 705 (61.3%) were not alone. Four hundred and twenty (36.5%) had either no children or one child, and 730 (63.5%) had two children or more. The two categories of occupational status were almost equal: 470 (40.9%) were farmers, artisans, manual workers or unemployed, and 489 (42.5%) were executives, middle professional group or clerical employees. Socio-economic status was low for 761 (66.2%) and high for 217 (18.9%) patients.

With regard to prevention practices, 355 (30.9%) women had no mammography in the 6 years before diagnosis, and 598 (52.0%) had had at least one; 194 (16.8%) patients had no gynaecological consultation in the 3 years before diagnosis, while 547 (47.6%) had had at least one for contraception, cervical smear or hormone therapy.

As shown in Table 1, univariate statistical analyses highlighted the fact that the socio-economic and surveillance characteristics of the 1150 women with primary breast cancer were significantly associated with the stage of cancer at diagnosis (stage 1 *vs* 2, 3 and 4 together), county of residence (*P*<0.001), age at diagnosis (*P*<0.001), way of life according to marital status (*P*<0.05), number of children (*P*<0.001), occupational status (*P*<0.001), socio-economic status (*P*<0.05), number of mammographies during the 6 years before diagnosis (*P*<0.001) and gynaecological prevention consultations during the 3 years before diagnosis (*P*<0.001). In multivariate logistic regression analysis, the factors associated with the probability of having breast cancer diagnosed stage 1 rather than stage 2, 3 or 4 were county of residence (Côte d'Or OR=2.0, 95% CI=[1.4–3.0]; Isère OR=2.6, 95% CI=[1.8–3.8]; Tarn OR=1.9, 95% CI=[1.3–2.9]; *P*<0.001), having two children or more (OR=1.9, 95% CI=[1.4–2.6]; *P*<0.001), occupational status ‘executives, middle professional group, clerical employees’ (OR=1.4, 95% CI=[1.02–1.8]; *P*<0.05), at least one mammography in the 6 years before diagnosis (OR=1.8 95% CI=[1.3–2.4]; *P*<0.001), and at least one gynaecological prevention consultation in the 3 years before diagnosis (OR=1.6, 95% CI=[1.2–2.2]; *P*<0.001).

Among the 1150 women with breast cancer in the study sample, 1135 (98.7%) were followed until June 2006: 815 (70.9%) women were still alive, 320 (27.8%) had died and 15 (1.3%) were lost to follow-up. Among the 1011 women without metastasis at the diagnosis, 515 (50.9%) were still alive without recurrence or metastasis, 99 (9.8%) were alive but with recurrence of their cancer, 158 (15.6%) were alive, but had developed metastases more than six months after diagnosis, while 239 (23.6%) had died. Among the 1135 patients followed-up, for 250 (22.0%) the cancer was detected, thanks to a symptom-free screening mammography, whereas for 755 (66.5%), the diagnosis mammography was performed because of clinical symptoms observed by the doctor or the woman herself, or if she was at a high risk for breast cancer. These data were unknown for 130 (11.5%) patients.

Table 2 shows 5- and 7-year survival rates with the log-rank test, and the results of the Cox multivariate analysis for overall survival. Five-year overall survival was 82.3% and 7-year overall survival was 75.0%.

The univariate survival analysis highlighted the fact that the county of residence (*P*=0.002), age at diagnosis (*P*<0.001), way of life (alone or not) (*P*=0.003), number of children (*P*=0.004), occupational status (*P*<0.001), socio-economic status (*P*=0.002), none or at least one mammography during the 6 years before diagnosis (*P*<0.001), none or at least one gynaecological prevention consultation during the 3 years before diagnosis (*P*<0.001) and cancer detected by screening mammography or not (*P*<0.001) were significantly associated with overall survival.

In multivariate analysis, the county of Isère (HR=0.7, 95% CI=[0.5–0.95]; *P*=0.024); age over 70 years at diagnosis (HR=2.0, 95% CI=[1.5–2.7]; *P*<0.001); occupational status including farmers, artisans, manual workers and unemployed (HR=1.4, 95% CI=[1.0–1.9]; *P*=0.050); no mammography in the 6 years before diagnosis (HR=1.8, 95% CI=[1.4–2.3]; *P*<0.001); no gynaecological prevention consultation in the 3 years before diagnosis (HR=1.8, 95% CI=[1.3–2.6]; *P*<0.001) and cancer detected on clinical symptoms and not by screening mammography (HR=2.2, 95% CI=[1.5–3.2]; *P*<0.001) were significantly associated with overall survival.

After adjustment for stage (Table 3), the factors that were significant were age above 70 years at diagnosis (HR=2.0, 95% CI=[1.5–2.6]; *P*<0.001), no mammography during the 6 years before diagnosis (HR=1.5, 95% CI=[1.1–2.0]; *P*=0.004), no gynaecological prevention consultation during the 3 years before diagnosis (HR=1.5, 95% CI=[1.1–2.2]; *P*=0.017) and cancer detected on clinical symptoms and not by screening mammography (HR=1.6, 95% CI=[1.05–2.3]; *P*=0.030).

In the same analysis, stage was significantly associated with overall survival, in comparison with stage T1 N0 M0: T2 and/or N+ (1, 2 or 3) M0 (HR=2.6, 95% CI=[1.9–3.6]; *P*<0.001) and T3 or T4 and/or M1 whatever the N (HR=7.1, 95% CI=[4.9–10.3]; *P*<0.001).

### Screening mammography as a prognostic factor of survival that is independent of age and cancer stage at diagnosis

Because screening mammography, as well as age and stage, is a prognostic factor (Table 2), we analysed it as a prognostic factor for overall survival within similar stages and age groups.

**Figure 1:**
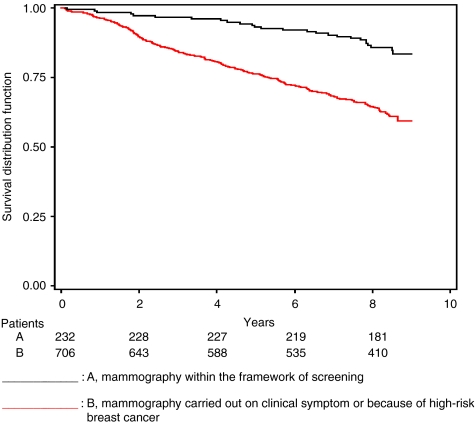
Overall survival distribution according to the circumstances of diagnosis mammography (Kaplan–Meier estimate).

Figure 1 describes the overall survival distribution according to the type of diagnosis mammography (screening or not). The results for 5-year overall survival and univariate analysis (log-rank test) by age group and by stage at diagnosis, according to the circumstances of the diagnosis mammography, are shown in Table 4.

Among the 253 women under 50 years, 18.2% had their diagnosis mammography within a screening framework and 81.8% on clinical symptoms or because of a high risk of breast cancer; 5-year survival for these two groups was 95.7 and 84.1%, respectively. Among the 514 women aged 50–70 years, the corresponding proportions were 33.5 and 66.5%, respectively, for those with 5-year survival of 93.6 and 87.1%, respectively, while among the 234 women aged over 70 years, these proportions were 13.2 and 86.8% with 5-year survival 93.7 and 66.5%, respectively. For each age group, patients for whom diagnosis was made in a screening mammography had significantly better overall survival than those who underwent mammography after onset of clinical symptoms, or because they had a high risk of breast cancer (*P*=0.005, *P*=0.011 and *P*<0.001, respectively, for the three age ranges).

Among the 343 women with stage 1 or 2 cancers (T1 N0 M0), 126 (36.7%) had their diagnosis mammography within a screening framework and 217 (63.3%) on clinical symptoms or because of a high risk of breast cancer; 5-year survival for the two groups was 94.4 and 93.1%, respectively; not significantly different. Among the 391 women with stage 3 cancer (T2 and/or N+ M0), the corresponding proportions were 13.3 and 86.7%, respectively, and 5-year survival for the two groups was 98.1 and 77.9%, respectively, a significant difference (*P*=0.002). Among the 91 women with stage 4 cancer (T3 or T4, N0 or +, and/or M1), these proportions were 5.7 and 94.3%, respectively, and 5-year survival for the two groups was 80.0 and 50.6%, respectively.

Survival analysis for the three subclasses of stage 3, T1 N+ M0, T2 N0 M0 and T2 N+ M0, provided significant results. Among the 133 women with stage T1 N+ M0, 26 (19.5%) had their diagnosis mammography within screening and 107 (80.5%) on clinical symptoms or because of a high risk of breast cancer, 5-year survival for the two groups being 100 and 78.5%, respectively (*P*=0.009); among the 128 women with stage T2 N0 M0, 17 (13.3%) had their diagnosis mammography within the framework of screening and 111 (86.7%) on clinical symptoms or because of a high risk of breast cancer, 5-year survival being 94.1 and 82.9%, respectively (*P*=0.046); among the 126 women with stage T2 N+ M0, eight (6.3%) had their diagnosis mammography within screening and 118 (93.7%) on clinical symptoms or because of a high risk of breast cancer, 5-year survival being 100 and 72.9%, respectively (*P*=0.833).

The number of patients who died, in the three subclasses of stage 3, was too low to make a survival analysis these results must be then considered as exploratory results.

## DISCUSSION

Many studies on breast cancer survival are hospital-based and/or use basic data on patient and tumour characteristics. As a complement to studies comparing screening mammography within a clinical trial (Nyström *et al*, 2002) or before and after a mammography screening programme (Tabar *et al*, 2003), our cancer registry data were collected from a well-defined French population and could thus be considered representative.

Our results support the major role of socio-economic data, surveillance characteristics and the circumstances of the diagnostic mammography on overall breast cancer survival, and confirm that they should be used to improve public health guidance for breast cancer.

Overall 5- and 7-year crude survival rates were 82.3 and 75.0%, respectively, for the whole sample. These results are in agreement with the 5-year relative survival rate of 81.3% published in the EUROCARE-3 study from French population-based registries diagnosed in 1990–1994 (Sant *et al*, 2003a). As highlighted in our study, survival varied significantly by county (Sant *et al*, 2003b). This is probably due to differences in the distribution of stage at diagnosis (Sant *et al*, 2003b) or to differences in the nature of the county (more urban or more rural) (Mitchell *et al*, 2006). We suggest that surveillance practices (a gynaecology consultation or a mammography during the years before diagnosis) as well as the circumstances of the mammography (screening or not) resulting from differences in the screening programmes in the counties may have influenced the stage at diagnosis. In addition, survival in the Bas-Rhin county was affected because it was included from 1995, when detection practices were not as good, and not 1997 as in other counties.

The surrogate way of life according to marital status (alone or not) was no longer significant in multivariate survival analyses, partly because of its correlation with age at diagnosis (older women are more often alone), surveillance practices (women not alone had better medical follow-up: more often went to a gynaecologist, had a mammography and their diagnosis mammography was more often performed in a screening framework) and number of children (such women are not alone). Furthermore, age and surveillance practices were independently associated with length of survival.

In contrast to another study (Kroman *et al*, 1998), parity was significant in multivariate analysis: as expected, it was associated having a partner, and with occupational and socio-economic status, but not in the same way; women in manual jobs and those with a low socio-economic status were more likely to have two or more children compared with those in the professions and with a high socio-economic status. However, the association with earlier stage at diagnosis was due to surveillance practices: women who had two or more children more often had gynaecology consultation or mammography than other women had. Finally, the number of children was also associated with age at diagnosis: women below 70 at diagnosis were more likely to have two or more children compared with those diagnosed at an older age.

In our study, the socio-economic variable ‘occupational’ was an independent prognostic factor of stage at diagnosis and of overall survival: the higher the occupational status, the better the stage and the longer the survival, as found in other studies (Thomson *et al*, 2001; Cross *et al*, 2002; Taylor and Cheng, 2003; Lagerlund *et al*, 2005; Downing *et al*, 2007). In France, it is difficult to obtain individual socio-economic data and to make comparisons with other varied studies. Our occupational subgroups probably encompassed varied social conditions, and our variable socio-economic status has no impact after adjustment. In Denmark, a population-based study of 28 765 cases (Dalton *et al*, 2006) showed that the risk of a high-risk breast cancer increased with reduced income, and with lower educational level. In the United States, SEER data showed that African-Americans had higher mortality even after adjustment for socio-economic factors (Grann *et al*, 2006). Even though stage at diagnosis explain part of the socio-economic differences in survival (Kaffashian *et al*, 2003; Lehto *et al*, 2006), future studies in France should use both patient data and area-based methods to quantify the effect of socio-economic variables on stage and survival more precisely; the role of financial difficulties, accessibility of health care and lifestyle need exploring in greater depth, as well as definitions of socio-economic profiles.

Surveillance and prevention practices were found to be major prognostic factors for survival. Having no mammography in the 6 years preceding diagnosis decreases 5-year survival by 18% and 7-year survival by more than 22% (*P*<0.001). Comparing women with one or more mammography to those who had none, we observed a reduced death risk hazard of 1.8 (95% CI=[1.4–2.3]; *P*<0.001) 9 years after diagnosis. Similarly, women who regularly consulted a gynaecologist had a survival benefit of 25%, 7 years after diagnosis, whereas those with none in the 3 years before diagnosis had an increased death risk hazard of 1.8 (95% CI=[1.3–2.6]; *P*<0.001). With respect to public health policy, these results suggest ways to improve survival.

We also found in multivariate analysis that asymptomatic mammographic detection, rather than mammography performed on clinical symptoms or because of a high risk of breast cancer, was a prognostic factor of survival. Patients who had their diagnosis mammography performed within the screening framework had better survival of 13.3% at 5 years, and 18.6% at 7 years (*P*<0.001), whereas the others who had mammography after the onset of clinical symptoms, or because they belonged to a high risk group, had an increased death risk hazard of 2.2 (95% CI=[1.5–3.2]; *P*<0.001). Other studies have also found that screening detected smaller tumours with a more favourable prognosis compared with those clinically detected (Fracheboud *et al*, 2004; Anttinen *et al*, 2006).

However, to our knowledge, mammography itself as a mode of detection has rarely been demonstrated as an independent prognostic factor in women who all had a diagnosis mammography, and in a population-based study, that is to say without any intervention or evaluation. In one study breast cancers detected initially by mammography, and after adjusting for stage, showed significantly higher survival than symptomatic cases (Gill *et al*, 2004); this persisted throughout the 9-year-period of follow-up, as in our study. Another showed that screening-detected tumours were associated with better 10-year disease-free survival than women those found outside screening (Joensuu *et al*, 2004). There could also be a reduction in advanced-stage tumours and an increase of more favourable low-stage tumours after several years of ongoing screening (McCann *et al*, 1998; Buiatti *et al*, 2003).

Whatever the stage at diagnosis, surveillance practices and the circumstances of the diagnosis mammography remain significant in multivariate analysis. Even though screening mammography offers better survival by way of a lower stage, unsurprisingly it is also an independent prognostic factor, whatever the stage. It is no surprise that the stage at diagnosis is a very strong prognostic factor for survival. Lastly, it is that in multivariate analysis, county of residence is no longer significant, these pre-diagnosis disparities disappearing with treatment.

Mass screening programmes are offered only to women aged 50–74 years. Breast cancer has increased in women under 50 in recent years, and as shown by Brenner and Hakulinen (2004), the protective effect of asymptomatic detection persists for decades. We thus studied screening mammography as a prognostic factor more closely. After adjusting for age, we showed that whatever their age, women who had their diagnosis mammography within the framework of screening had better survival more than 9 years after diagnosis (*P*=0.005, *P*=0.011 and *P*<0.001, respectively).

We could not demonstrate any difference between screening-detected cancers and clinically detected for stages 1, 2 and 4; but for stage 3 (T2 and/or N+ M0), we found much better survival with tumours detected by screening mammography (*P*=0.002). This benefit is apparent for tumours larger than 2 cm, which is unlikely to be a result of lead time or length bias, but these subclasses seem to be the more responsive to mass screening, and it may be thought that when the tumour is small, whatever the extent of node invasion, survival is better. However, due to sample size limitations in the subgroups, these results should be interpreted carefully and validated on a large sample.

## Figures and Tables

**Table 1:** Patients' socio-economic and surveillance characteristics, univariate and multivariate logistic regression analysis of prognostic factors for stage at diagnosis (stage 1 *vs* stages 2, 3 and 4 together)

		**Univariate analysis (logistic regression)^a^**		**Multivariate analysis (logistic regression)^a^**	
	***n*=1150 (%)**	**Odds ratio [95% CI]**	***P*-value**	**Odds ratio [95% CI]**	***P*-value**
*County of residence*				1	Doubs and Bas-Rhin together
Doubs	248 (21.6)	1			
Bas-Rhin	193 (16.8)	1.2 [0.7–2.0]	<0.001		
Côte d'Or	228 (19.8)	2.0 [1.3–3.1]		2.0 [1.4–3.0]	
Isère	269 (23.4)	2.5 [1.6–3.7]		2.6 [1.8–3.8]	<0.001
Tarn	212 (18.4)	1.6 [1.03–2.5]		1.9 [1.3–2.9]	
					
*Age at diagnosis*					
<50 years old	280 (24.3)	1.7 [1.2–2.6]			
50–70 years old	569 (49.5)	2.0 [1.5–2.0]	<0.001	NS	
>70 years old	292 (25.4)	1			
Unknown	9 (0.8)				
					
*Marital status*					
Alone	354 (30.8)	1	<0.05	NS	
Not alone	705 (61.3)	1.4 [1.04–1.9]			
Unknown	91 (7.9)				
					
*Number of children*					
0 or 1	420 (36.5)	1	<0.001	1	
2 or more	730 (63.5)	1.6 [1.2–2.2]		1.9 [1.4–2.6]	<0.001
					
*Occupational status*					
Farmers, artisans, manual workers, unemployed	470 (40.9)	1	<0.001	1	
Executives, middle professional group, employees	489 (42.5)	1.7 [1.6–2.4]		1.4 [1.02–1.8]	<0.05
Unknown	191 (16.6)				
					
*Socio-economic status*					
Low	761 (66.2)	1	<0.05	NS	
High	217 (18.9)	1.4 [1.02–2.0]			
Unknown	172 (14.9)				
					
*Mammographies during the 6 years before diagnosis*					
None	355 (30.9)	1		1	<0.001
One or more	598 (52.0)	2.4 [1.9–3.0]	<0.001	1.8 [1.3–2.4]	
Unknown	197 (17.1)				
					
*Gynaecological prevention consultation during the 3 years before diagnosis*					
None	194 (16.8)	1	<0.001	1	
One or more	547 (47.6)	2.4 [1.8–3.2]		1.6 [1.2–2.2]	<0.001
Unknown	409 (35.6)				

Abbreviations: CI=confidence interval; NS=not significant.

a Unknown data were not used for the statistical analyses.

**Table 2:** Univariate and multivariate analyses of overall survival according to socio-economic and surveillance characteristics (*n*=1138)

	**Overall survival (%)**			**Cox multivariate analysis**	
	**5-year survival (%)**	**7-year survival (%)**	**Univariate analysis log-rank *P*-value**	**Hazard ratio [95% CI]**	***P*-value**
*County of residence*				1	Doubs and Bas-Rhin together
Doubs	82.4	74.4			
Bas-Rhin	79.2	75.5			
Côte d'Or	82.2	78.2	0.002	0.9 [0.7–1.3]	0.660
Isère	88.9	79.6		0.7 [0.5–0.95]	0.024
Tarn	76.7	66.2		1.0 [0.7–1.4]	0.963
					
*Age at diagnosis*					
<50 years old	86.2	82.3		1.1 [0.8–1.6]	0.441
50–70 years old	87.9	83.1	<0.001	1	
>70 years old	67.6	52.4		2.0 [1.5–2.7]	<0.001
					
*Marital status*					
Alone	78.5	69.9	0.003	0.9 [0.7–1.2]	0.557
Not alone	84.2	78.1		1	
					
*Number of children*					
0 or 1	78.3	69.6	0.004	1.2 [0.9–1.5]	0.241
2 or more	84.6	78.2		1	
					
*Occupational status*					
Farmers, artisans, manual workers, unemployed	77.4	69.4	<0.001	1.4 [1.0–1.9]	0.050
Executives, middle professional group, clerical employees	88.2	83.0		1	
					
*Socio-economic status*					
Low	81.9	74.0	0.002	1.0 [0.7–1.5]	0.991
High	89.7	84.5		1	
					
*Mammographies during the 6 years before diagnosis*					
None	72.0	62.9	<0.001	1.8 [1.4–2.3]	<0.001
One or more	90.0	85.3		1	
					
*Gynaecological consultation*					
None	73.7	60.5	<0.001	1.8 [1.3–2.6]	<0.001
One or more	89.5	85.4		1	
					
*Cancer detected by screening mammography*					
No	80.7	72.6	<0.001	2.2 [1.5–3.2]	<0.001
Yes	94.0	91.2		1	

Abbreviation: CI=confidence interval.

**Table 3:** Univariate and multivariate analyses of overall survival according to socio-economic and surveillance characteristics plus breast cancer stage at diagnosis (*n*=1138)

	**Overall survival (%)**			**Cox multivariate analysis**	
	**5-year survival (%)**	**7-year survival (%)**	**Univariate analysis log-rank *P*-value**	**Hazard ratio [95% CI]**	***P*-value**
*County of residence*				1	Doubs and Bas-Rhin together
Doubs	82.4	74.4			
Bas-Rhin	79.2	75.5			
Côte d'Or	82.2	78.2	0.002	0.9 [0.6–1.2]	0.401
Isère	88.9	79.6		0.7 [0.5–1.0]	0.068
Tarn	76.7	66.2		1.1 [0.8–1.4]	0.738
					
*Age at diagnosis*					
<50 years old	86.2	82.3		1.1 [0.8–1.6]	0.576
50–70 years old	87.9	83.1	<0.001	1	
>70 years old	67.6	52.4		2.0 [1.5–2.6]	<0.001
					
*Marital status*					
Alone	78.5	69.9	0.003	0.9 [0.7–1.2]	0.521
Not alone	84.2	78.1		1	
					
*Number of children*					
0 or 1	78.3	69.6	0.004	1.0 [0.8–1.3]	0.754
2 or more	84.6	78.2		1	
					
*Occupational status*					
Farmers, artisans, manual workers, unemployed	77.4	69.4	<0.001	1.3 [0.9–1.8]	0.106
Executives, middle professional group, clerical employees	88.2	83.0		1	
					
*Socio-economic status*					
Low	81.9	74.0	0.002	1.1 [0.8–1.7]	0.568
High	89.7	84.5		1	
					
*Mammographies during the 6 years before diagnosis*					
None	72.0	62.9	<0.001	1.5 [1.1–2.0]	0.004
One or more	90.0	85.3		1	
					
*Gynaecological consultation*					
None	73.7	60.5	<0.001	1.5 [1.1–2.2]	0.017
One or more	89.5	85.4		1	
					
*Cancer detected by screening mammography*					
No	80.7	72.6	<0.001	1.6 [1.05–2.3]	0.030
Yes	94.0	91.2		1	
					
*Stage TNM*					
T1 N0 M0	93.8	89.2		1	
T2 and/or N+ M0	80.7	71.7	<0.001	2.6 [1.9–3.6]	<0.001
T3 or T4 and/or M1	44.6	33.9		7.1 [4.9–10.3]	<0.001

Abbreviation: CI=confidence interval.

**Table 4:** Screening mammography as a prognostic factor of survival, by age and by stage at diagnosis: descriptive, 5-year overall survival rate and overall survival in univariate analysis

**Circumstances of diagnosis mammography**	***n* (%)**	**5-year overall survival (%)**	**Univariate analysis *P*-value (log rank)**
*Age at diagnosis*			
*<50 years old*	*n*=253		
Screening mammography	46 (18.2)	95.7	0.005
On clinical symptom or high-risk	207 (81.8)	84.1	
			
*From 50 to 70 years old*	*n*=514		
Screening mammography	172 (33.5)	93.6	0.011
On clinical symptom or high-risk	342 (66.5)	87.1	
			
*>70 years old*	*n*=234		
Screening mammography	31 (13.2)	93.7	<0.001
On clinical symptom or high-risk	203 (86.8)	66.5	
			
*Cancer stage at diagnosis*			
*1 and 2: T1 N0 M0*	*n*=343		
Screening mammography	126 (36.7)	94.4	0.417
On clinical symptom or high-risk	217 (63.3)	93.1	
			
*3: T2 and/or N+ M0*	*n*=391		
Screening mammography	52 (13.3)	98.1	0.002
On clinical symptom or high-risk	339 (86.7)	77.9	
			
*T1 N+ M0*	*n*=133		
Screening mammography	26 (19.5)	100	0.009
On clinical symptom or high-risk	107 (80.5)	78.5	
			
*T2 N0 M0*	*n*=128		
Screening mammography	17 (13.3)	94.11	0.046
On clinical symptom or high-risk	111 (86.7)	82.9	
			
*T2 N+ M0*	*n*=126		
Screening mammography	8 (6.3)	100	0.833
On clinical symptom or high-risk	118 (93.7)	72.9	
			
*4: T3 or T4 and/or M1*	*n*=88		
Screening mammography	5 (5.7)	80.0	0.159
On clinical symptom or high-risk	83 (94.3)	50.6	
